# General practitioners’ attitudes towards patients with poorly controlled type 2 diabetes: a qualitative study

**DOI:** 10.1186/s12875-018-0751-4

**Published:** 2018-05-02

**Authors:** Anja Wollny, Michael Pentzek, Oliver Rudolf Herber, Heinz-Harald Abholz, Jürgen in der Schmitten, Andrea Icks, Stefan Wilm, Elisabeth Gummersbach

**Affiliations:** 1 Institute of General Practice, University Medical Center Rostock, Rostock, Germany; 2 Institute of General Practice (ifam), Medical Faculty, Heinrich-Heine University Düsseldorf, Werdener Str. 4, 40227 Düsseldorf, Germany; 3 Institute for Health Services Research and Health Economics, Medical Faculty, Heinrich-Heine University Düsseldorf, Düsseldorf, Germany

**Keywords:** Diabetes mellitus, Type 2, Physician-patient relations, General practice, Attitude of health personnel, Qualitative research

## Abstract

**Background:**

Patients with type 2 diabetes mellitus (T2DM) with poor glycaemic control can represent a challenge from the perspective of the general practitioner (GP). Apart from patient-sided factors, the understanding of GPs’ attitudes may provide ideas for improved management in these patients. The aim of this study is to reveal attitudes of GPs towards T2DM patients with poor metabolic control.

**Methods:**

Qualitative research in German general practice; 20 GPs, randomly chosen from participants of a larger study; in-depth narrative interviews, audio-recorded and transcribed; inductive coding and categorisation in a multi-professional team; abstraction of major themes in terms of attitudinal responses.

**Results:**

1) Orientation on laboratory parameters: GPs see it as their medical responsibility to achieve targets, which instil a sense of security. 2) Resignation: GPs believe their efforts are in vain and see their role as being undermined. 3) Devaluation of the patient*:* GPs blame the “non-compliance” of the patients and experience care as a series of conflicts. 4) Fixed role structure: The expert GP on the one hand, the ignorant patient on the other. 5) Solidarity with the patient: GPs appreciate a doctor-patient relationship in terms of partnership.

**Conclusions:**

The conflict GPs experience between their sense of duty and feelings of futility may lead to perceptions such as personal defeat and insecurity. GPs (and patients) may benefit from adjusting the patient-doctor relationship with regard to shared definitions of realistic and authentic goals.

## Background

A more detailed understanding of GPs’ attitudes is the basis for achieving both, higher job satisfaction and better healthcare. The theoretical rationale for this is a cascade of well described psychological principles: In a tripartite model [1], attitudes are composed of three elements: cognitive (beliefs, evaluation), affective (emotions), and conative (behavioural intentions). Attitudes have been shown to have an effect on observable behaviour [2] – a fact that underlies several theories of explaining or changing behaviour [3].

In T2DM, practitioners’ perceptions of patients and their conditions have been shown to have significant impact on the course of a consultation [4, 5]. Thus, the observable behaviour of practitioners (i.e. doctor-patient communication, e.g. in terms of the development of therapeutic aims) can be influenced by the practitioners’ attitudes towards a patient [6, 7]. Ultimately, professional behaviour (i.e. the GP’s communication style and technique) exerts its effects on patient outcomes by several routes [8].

In spite of efforts to improve the treatment of type 2 diabetes mellitus (T2DM), a relevant proportion of patients with T2DM still remains in poor metabolic control [9]. In Germany, more than two thirds of T2DM patients take part in the corresponding DMP (disease management program), including three-monthly controls of metabolic and physical parameters [10]. For general practitioners (GPs), poorly controlled diabetes is among the independent factors of higher perceived complexity in patient care [11]. The reasons for this are not fully understood. Previous studies on GPs’ attitudes towards diabetes have focused on GPs’ generalized justifications for poor metabolic control, with a strong emphasis on GPs’ patient-sided attributions like lack of knowledge or compliance [12, 13].

Andersen already suggested in 1998 that structured care programs for people with diabetes would benefit from more qualitative research on the attitudes held by professional carers [14]. With a qualitative methodological approach (narrative interviews about individual patients), we aimed for a differentiated description of physician-sided attitudinal aspects of caring for patients with poorly controlled diabetes. The results will be relevant for changing clinical practice, constructing educational interventions and composing guidelines, as these will benefit from reflecting and considering GPs’ attitudes and stereotypes.

## Methods

The study presented here is part of a mixed methods project focusing on characteristics common among poorly controlled T2DM patients, which is described in more detail elsewhere [15]. Sixty-seven general practitioners (48.9% response) from the region of North Rhine Westphalia took part. From these, we randomly selected 20 GPs for the qualitative study part. All accepted the invitation and provided informed consent.

### Data collection

In the narrative technique [16], the narration of the interviewee is of vital relevance and contains the most meaningful material. To stimulate such narration, asking for reports about personal relevant and memorable experiences are most adequate. Individual patients are personally relevant to a GP, rather than asking for an abstract concept (e.g., ‘poor metabolic control’). The narrative stimulus and internal probes were developed in four sessions within the research team, considering both, narrative interview methodology as well as wording and expertise of practicing GPs. The resulting interview protocol was pretested by the interviewers in two supervised interviews with GPs and refined in the research team. The initial narrative stimulus was: “I am interested in your patients with diabetes. I would now ask you to tell me something about a type 2 diabetes patient who is, according to your opinion, in poor control. Just think of a poorly controlled patient who comes to mind spontaneously. What’s it like with this patient? You can take as much time to tell as you want.” After presenting this patient, the GP was asked to talk about a second T2DM patient which had been previously selected from the patient list for inclusion in the overall mixed methods project: “You have reported to the project both well-controlled and poorly controlled diabetes patients. Of these, I have selected two. First of all, I would like to ask you to tell me something about the patient [XY] who is one of the poorly controlled patients.” To stimulate the interview after the initial narrations, probing after each case focussed on detailing aspects that had been mentioned by the GP in his/her narration (e.g., specific situations like single consultations, persons like patient’s relatives, personal evaluations like justifications or emotional reactions, time periods like crises in the doctor-patient relation). Two researchers were trained in in-depth narrative interviewing [17] by AW in four 2-h training sessions including roleplays. Interviewers were supervised throughout the study by AW and SW. Interviewers were a male psychologist (MP, 13 interviews) and a female GP (EG, 7 interviews). Both are experienced in qualitative research and neither were acquainted with any of the interviewed GPs. The interviews were carried out in the GPs’ own surgeries at their convenience and on average lasted 47 min (28 to 80 min). All interviews were audio-taped, transcribed verbatim, and pseudonymized. Field notes were taken by the interviewers during and after the interviews.

### Data analysis

The analyses started after all interviews had been completed and followed an iterative cross-case approach in a multidisciplinary team of 11 physicians and researchers. As the objective was not a new attitude theory, we applied conventional (i.e., inductive) content analysis [18] to create patterns of attitudinal aspects based on the theoretical framework described above (the tripartite attitude model [1]). According to Moser and Korstjens [19], we combined several techniques. All steps were performed with investigator triangulation in a multidisciplinary team to apply different perspectives to the data and thereby strengthening the credibility of the results [20]. Inductive coding sessions were guided by the question, which beliefs, emotions, and intentions can be found in the reports of GPs about their T2DM patients in poor metabolic control. Six of the twenty interviews were chosen for inductive coding, selected for contrasting GP/patient gender combinations and content of the interviewer memos. Twelve inductive coding sessions (2 per interview, 60–120 min each) were conducted in varying constellations with a median/mean of 5 participants out of the 11 members of the analysis team (3–10 per session; at least one physician and one researcher in each session; AW and EG coordinated the analyses and participated in each session). Results of these sessions were recorded in protocols; a practicing GP (EG) and a health care researcher with experience as physician assistant (AW) differentiated codes by constant comparison of all case reports within and across the 6 interviews. Based on this and the theoretical attitude model, codes were summarised in categories. Using these categories, EG and AW continued with axial coding of the remaining 14 interviews. The final interpretation was guided by principles of general practice (e.g. shared decision making) as well as clinical GP experience with the aim of constructing attitudinal patterns (i.e. meaningful combinations of single attitudinal aspects (cognitive/affective/conative)), resulting in five major themes [21]. The latter were reflected in the whole analysing team (11 members), discussed and refined, always referring back to the transcripts.

## Results

Participant characteristics: 14 male, 6 female GPs, mean age 53.5 years (SD 7.2), mean years in practice 17.3 (SD 6.6), mean number of T2DM patients in GP practice 140.9 (SD 84.9), maximum HbA1c (glycated haemoglobin) 12.1% (SD 1.6), mean HbA1c of patients spontaneously mentioned by GPs in the interviews 9.2 (SD 1.8).

Five major themes describe patterns of GPs’ attitudes (see Table 1).

**Table 1:** GPs’ attitudes towards patients with poorly controlled type 2 diabetes mellitus

Theme	Description
Confidence vs. pressure through orientation on laboratory parameters	GPs see it as their medical responsibility to achieve targets which instil a sense of security, but also pressure.
Low self-efficacy and feelings of resignation	GPs believe their efforts are in vain and see their role as being undermined.
Lack of understanding and devaluation of the patient	GPs blame the “non-compliance” of the patients and experience care as a series of conflicts.
Establishment of a fixed role structure	The expert GP on the one hand, the ignorant patient on the other.
Showing solidarity with the patient	GPs appreciate a doctor-patient relationship in terms of partnership.

### Confidence vs. pressure through orientation on laboratory parameters

For the GPs, it is part of their medical responsibility to achieve the targets defined by the DMP (disease management program) diabetes, triggering two possible emotions: On the one hand, these targets (e.g. remaining below a maximum HbA1c) seem to bring about a sense of security, which is highly valued by the GPs.

*“But like I said, that’s basically the crunch, where I actually say: ‘You’ve got to get under 7.’ See? But that’s basically -erm- a problem. He always manages it then, then it always goes down a bit to 7.3. So the trend is pretty good again. But like I said, erm, - we find here that people when they’ve been on a course, they get very good, they get really good HbA1c levels. Under 7.5, sometimes even under 7. And I’d say about 10% stick to it […]”* (GP #58)

On the other hand, pressure to reach these targets results in repeated and exhausting attempts to explain potential long-term consequences (e.g. by means of patient education, fear appeals or admonitions).

GPs describe the “referral to a diabetes expert” as a kind of last resort, once the aforementioned approaches have failed.

*“Perhaps he will have to be referred to a diabetes specialist, after all, with the instruction to give him insulin, if there’s no improvement with the HbA1c, with the fasting blood sugar level; we do need to think about protecting his kidneys, perhaps switching him to insulin a bit earlier than would normally be done.”* (GP #13)

After all their efforts, GPs expect their patients to comply with what they should have learned. At the same time, they realize that these hopes are often in vain. Responsibility for this failure is generally attributed to the patients.

### Low self-efficacy and feelings of resignation

Behind the facade of professionalism, propped up by the pursuit of fixed target values, GPs feel personally affected by conflicts with their patients. Unable to reach their aims they suffer from feelings of failure and defeat.

*“But like I said we ...despite us trying absolutely everything, she’s always ending up in hospital because of hypertension or hyperglycaemia; but all, all attempts to manage her diabetes have just failed and up to now haven’t been as successful as we really would have liked and now it’s basically all too late, all too late.”* (GP #110)

The inability to change things undermines the GPs’ self-perceived role and self-efficacy as doctors and can lead to feelings of helplessness. The “non-compliance” of patients, which is repeatedly mentioned and deplored by the GPs, is seen as a personal insult. Furthermore, these feelings of failure may culminate in resignation and flagging effort.

*“And I went into practice with the necessary enthusiasm. […] with good ideas. And then when over the years you realise that you are basically talking to a brick wall..., with lots of patients – but please no generalisations. That’s frustrating, definitely. Then, at some point, your enthusiasm for getting the patient on the right track begins to wane.”* (GP #115)

In contrast to this, we found other GPs compensating for their failure by intensifying their engagement (e.g. by sharing their private telephone numbers for constant availability).

### Lack of understanding and devaluation of the patient

General practitioners speak about some T2DM patients as self-reliant people who cannot be forced into a certain treatment concept. The GPs claim to know what is best for their patients, but have a difficult time to understand why their advice is not being followed.

*“...and the weight loss isn’t noticeable, either, the BMI stays at 40. Then it’s ... difficult. Very difficult. In that case it’s certainly not the doctors who are to blame for the badly controlled diabetes. In that case I’d look first at what the patient wasn’t doing right.”* (GP #115)

In return for their own dedication, GPs expect patients to change their behaviour while knowing this is unlikely to happen. The patient’s behaviour is not seen as an indication of autonomy but is often attributed to a lack of insight or simple-mindedness.

*“In any case, she can’t cope with the details. [...] Some people just are a bit simple-minded. […] ok, this woman, for example, comes with an even worse HbA1c level, about 10. And, erm, so there is no downward trend but in fact more an increasing one. And, eh, everything we’ve talked about has actually gone into thin air. And, erm, then I do get annoyed that I’ve taken all this trouble. That I can’t sort of not care about it. I’d like that sometimes. That I don’t care. She’s not cooperating. So that’s it. That’s sort of fate.”* (GP #8)

Care for these patients is described as a struggle and characterised by conflicts. Some of these patients are seen as prototypes *(“the typical adipose diabetic”*), others as atypical extreme cases.

*“And he can’t stop eating, […]. He’s simply obsessed, erm, it’s a passion for him, eh? […] In that house, then, everything revolves around food. Food is, erm, the factor that keeps them all together.”* (GP #25)

### Establishment of a fixed role structure

On the one hand, there is the expert GP making strenuous efforts on the patient’s behalf; on the other side, there is the ignorant or unqualified patient who is in denial and refuses to follow advice.

*“Apart from that, a very nice talkative patient who’s always reasonable. So, you think that he would have understood it. And, erm, he also knows a lot. And that’s what’s so bad about the whole thing, And, erm,- but it’s never been any use, eh? To give a sermon to him - and all for nothing, eh? And even now it’s like that he, erm, - as he says so nicely - is doing something about it, erm, but at the same time he doesn’t come to see us regularly.”* (GP #194)

Some GPs perceive themselves as victims of patients who prevent them from exercising their medical expertise. The patients are seen as kind of culprits who bring about problematic situations as a result of their specific conduct.

*“And I, erm, asked him to show me his booklet which, yet again, he didn’t have with him, but he said his levels were all ok. And besides it was all-in-all an improvement. And it’s true in a way [...] And that is really frustrating. Working with patients like this. [...] With some patients, though, you can, in certain conditions, achieve something by trying to frighten them. You see, fear can sometimes help a bit.”* (GP #132)

The GPs’ ongoing attempts to “convert” the patient, using perseverative discussions despite being aware of their futility, are reminiscent of the predicament known from carers of addicts. The fixed role structure serves to reinforce GPs’ self-confidence and to relieve pressure. In their role as “preachers” they try to “absolve” patients of their “sins” (e.g. “poor” eating habits).

*“He’s been on all the educational courses and he is fully aware of the consequences. I lay them all on the table for him time after time and tell him that he can’t go on like this but he’s got a thousand excuses. […] very busy at work. Problems with his wife. Erm ... then his addiction behaviour with curried fried sausage (Currywurst) [laugh]. […] Yes, that’s it, that’s what he orders every time. So, you don’t get anywhere with him there. […] He says he knows about it: ‘I’ve been to all the courses, I’ve been in the hospital at [city] thousands of times’, he’s been there, I think, six or seven times. [...] Well, you can’t educate him any better. You can’t improve him either; that is, you can only keep on talking and talking, that’s how it is. […] but most of the time it’s pointless.”* (GP #170)

Interestingly, a special dispensation is granted to patients who are academics: GPs show more understanding of their behaviour and tend to justify inappropriate self-care.

### Showing solidarity with the patient

Some GPs claim to have developed strategies to cope with conflicts they have with poorly controlled T2DM patients. In order to maintain their capacity to act, they have learned to protect themselves. A doctor-patient relationship in terms of partnership is desired but cannot be realised in many cases.

*“But apart from that she’s happy, this lady. I’m not frustrated either [laughs]. I mean, yeah, it’s sad that her HbA1c levels don’t go down, eh? But I always wonder “Shouldn’t you say something again, shouldn’t you put pressure on her again”, but will she then be any happier, eh? Or, sometimes I do it again, you know, increase her insulin. And then you see it doesn’t do any good. That’s the situation in which you find yourself, eh? [...] She’s well. In quotation marks, you see? With a few little limitations, but she feels, she feels well like this, quite rightly, eh? [...] What are you supposed to do?”* (GP #194)

But in some cases, the autonomy of the patient is accepted. With this approach, a partnership between GP and patient becomes possible to a certain extent. But there still remains incredulity and resistance with regard to the possibility and effectiveness of adopting a strategy of “shared decision making”.

*“So, she does it a bit, not according to a plan, not according to instructions [...], not according to a prescribed plan. And it’s going reasonably well for her. And I can’t interfere either, eh? She is so well versed with her sugar problem and has been on so many courses. So, she says to me: “You can’t tell me much. I’m already doing it and I have to go with how I feel. And I also know how it works.” And, erm, so then her readings don’t contradict her, especially as they weren’t any better when she was strictly controlled.”* (GP #122)

The analysis reveals that GPs would prefer to give up the constant struggle with their patients and, instead, work together with them. Despite signalling this in the interviews, they seem to continue sticking to formal outcomes.

## Discussion

This study explores in depth the beliefs, emotions, and intentions that physicians report in relation to patients with poorly controlled T2DM. Our analysis of 20 narrative interviews with GPs has revealed five major themes and can be summarised as follows*.*

The targets, as defined by the German structured disease management program (DMP), provide a stable framework which gives GPs confidence; on the other hand, GPs feel under pressure by these targets. The failure to reach these targets can provoke further emotional reactions like personal defeat or insecurity; GPs are torn between their sense of responsibility and a feeling of futility. This can result in decreasing interest and engagement, possibly culminating in abandoning the patient. GPs perceive a damage that has been done to their sense of professionalism and self-efficacy as a doctor. They try to compensate this damage by continuously striving for the standards set down in the DMP recommendations. The constant “preaching” in order to reach these standards becomes an end in itself because their efforts are often in vain. Forced to give up in the end they blame failure on the patient’s shortcomings. Sometimes, however, GPs accept their patient’s autonomy.

Qualitative studies dealing with how general practitioners manage patients with T2DM were often carried out using focus groups [22]. These studies primarily provide information on GPs’ justification of problems and attributions to patient factors (esp. lack of patient knowledge and compliance) [13]. In line with our results, feelings of frustration have also been reported [23]; our results especially differentiate these emotional aspects. Feelings of resignation and low self-efficacy are likely to hinder professional primary care for T2DM patients with poor metabolic control. In this aspect, there are parallels to doctors’ attitudes towards substance abuse [24], where professional distance is one important prerequisite for the transfer of responsibility to the patient, adopting a more advisory role of the GP [25].

Our results further adumbrate some of the GP-sided difficulties in realizing patient-centred consultations and shared decision making (SDM). Practitioners and patients with diabetes may disagree about therapeutic aims [26]. On the other hand, T2DM patients prefer a patient-centred approach [12], and also doctors acknowledge the positive effect of patient-centeredness, but find it difficult to adopt [27]. Our analysis provides an understanding how a patient-centred approach and SDM, here in particular in the form of shared development of therapeutic aims, may not only be in line with current medico-ethical and legal frameworks. Rather, it may also make a large difference both for job satisfaction on the side of physicians, and for providing satisfying care in the perception of patients with poorly controlled T2DM.

Physicians (like everybody else) are influenced by stereotypes, i.e. categorical information [28, 29]. It is possible that parts of our results point to a possible stereotype of the “complex diabetic patient” which to evaluate would warrant further research.

All participating GPs took part in the DMP Diabetes (as 80% of Germans GPs do). Thus, our results may not be transferable to non-DMP GPs. Half the GPs invited took part in the mixed methods study. Despite this relatively high participation rate it cannot be excluded that these GPs may have an above average interest in diabetes and/or related research, or over-represent certain attitudes.

The strength of the study lies in the narrative and multidisciplinary approach. Other than semi-structured and group methods, where evaluation, description, and argumentation are among the typical sorts of response, narrative interviews focus on individual cases, produce ad-hoc narratives and are capable of generating a deeper understanding of the reasons why people feel, think and act in a particular way [17].

## Conclusions

The conflict GPs feel between their sense of duty and feelings of futility may lead to reactions such as personal defeat, insecurity, and a hurt sense of professionalism and self-efficacy. In accordance with modern goal-setting strategies [30, 31], accepting patients’ autonomy and shifting goals from HbA1c-limits to shared definitions of realistic and patient-relevant goals may be useful in alleviating GPs’ tensions [32].

## Abbreviations

DMP: Disease management program
GP: General practitioner
HbA1c: Glycated haemoglobin
SDM: Shared decision making
T2DM: Type 2 diabetes mellitus

## References

[1] Hogg M, Vaughan G (2005). Social psychology.

[2] Fazio RH, Roskos-Ewoldsen DR. Acting as we feel: when and how attitudes guide behavior. In: Shavitt S, Brock TC, editors. Persuasion: psychological insights and perspectives. Needham Heights MA: Allyn Bacon; 1994. p. 71–93.

[3] Cameron KA (2009). Theories in health communication research. A practitioner’s guide to persuasion: an overview of 15 selected persuasion theories, models and frameworks. Patient Educ Couns.

[4] Freeman J, Loewe R (2000). Barriers to communication about diabetes mellitus. Patients’ and practitioners’ different views of the disease. J Fam Pract.

[5] Olivarius N, Palmvig B, Helms Andreasen A, Thorgersen J, Hundrup C (2005). An educational model for improving diet counselling in primary care. A case study of the creative use of doctors’ own diet, their attitudes to it and to nutritional counselling of their patients with diabetes. Patient Educ Couns.

[6] Street RL, Gordon H, Haidet P (2007). Practitioners’ communication and perceptions of patients: is it how they look, how they talk, or is it just the doctor?. Soc Sci Med.

[7] An PG, Rabatin JS, Manwell LB, Linzer M, Brown RL, Schwartz MD for the MEMO Investigators (2009). Burden of difficult encounters in primary are: data from the minimizing error, maximizing out-comes study. Arch Intern Med.

[8] Street RL, Makoul G, Arora NK, Epstein RM (2009). How does communication heal? Pathways linking clinician-patient communication to health outcomes. Patient Educ Couns.

[9] Nicholas J, Charlton J, Dregan A, Gulliford MC (2013). Recent HbA1c values and mortality risk in type 2 diabetes. PLoS One.

[10] Altenhofen L, Hagen B, Hansen L, Günster C, Klose J, Schmacke N (2010). Ergebnisse zur DMP-Umsetzung in der region Nordrhein. Versorgungs-report 2011: Schwerpunkt Chronische Erkrankungen.

[11] Grant RW, Ashburner JM, Hong CS, Chang Y, Barry MJ, Atlas SJ (2011). Defining patient complexity from the primary care Physician's perspective. Ann Intern Med.

[12] Jeavons D, Hungin APS, Cornford CS (2006). Patients with poorly controlled diabetes in primary care: healthcare clinicians’ beliefs and attitudes. Postgrad Med J.

[13] Moreau A, Aroles V, Souweine G (2009). Patient versus general physician perception of problems with treatment adherence in type 2 diabetes. Eur J Gen Pract.

[14] Anderson R, Robins L (1998). How do we know? Reflections on qualitative research in diabetes. Diabetes Care.

[15] Wilm S, Abholz HH, Gummersbach E, Icks A, Pentzek M. Patients with poorly regulated type 2 diabetes – health psychological characterization. Diabetologe. 2014;10:200–206. [German].

[16] Jovchelovitch S, Bauer MW, Bauer MW, Gaskell G (2000). Narrative interviewing. Qualitative research with text, image and sound: a practical handbook.

[17] Chase SE, Lincoln YS, Denzin NK (2003). Taking narrative seriously: consequences for method and theory in interview studies. Turning points in qualitative research.

[18] Hsieh HF, Shannon S (2005). Three approaches to qualitative content analysis. Qual Health Res.

[19] Moser A, Korstjens I. Practical guidance to qualitative research. Part 3: Sampling, data collection and analysis. Eur J Gen Pract. 2017; doi: 10.1080/13814788.2017.1375091. 10.1080/13814788.2017.1375091 PMC5774281 29199486

[20] Korstjens I, Moser A. Practical guidance to qualitative research. Part 4: Trustworthiness and publishing. Eur J Gen Pract. 2017; doi: 10.1080/13814788.2017.1375092. 10.1080/13814788.2017.1375092 PMC8816392 29202616

[21] Elo S, Kyngäs H (2008). The qualitative content analysis process. J Adv Nurs.

[22] Brown J, Harris S, Webster-Bogaet S, Wetmore S, Faulds C, Steward M (2002). The role of patient, practitioner and systemic factors in the management of type 2 diabetes mellitus. Fam Pract.

[23] Wens J, Vermeire E, Van Royen P, Sabbe B, Denekens J (2005). Practitioners’ perspectives of type 2 diabetes patients’ adherence to treatment: a qualitative analysis of barriers and solutions. BMC Fam Pract.

[24] Ketterer F, Symons L, Lambrechts MC (2014). What factors determine Belgian general practitioners’ approaches to detecting and managing substance abuse? A qualitative study based on the I-change model. BMC Fam Pract.

[25] Nabitz U, van den Brink W, Walburg J (2005). A quality framework for addiction treatment programs. Addict Behav.

[26] Heisler M, Vijan S, Anderson RM, Ubel PA, Bernstein SJ, Hofer TP (2003). When do patients and their practitioners agree on diabetes treatment goals and strategies, and what difference does it make?. J Gen Intern Med.

[27] Beverly E, Hultgren B, Brooks K, Ritholz M, Abrahamson MJ, Weinger K (2011). Understanding physicians’ challenges when treating type 2 diabetic patients’ social and emotional difficulties. Diabetes Care.

[28] Chapman EN, Kaatz A, Carnes M (2013). Physicians and implicit bias: how doctors may unwittingly perpetuate health care disparities. J Gen Intern Med.

[29] Galinsky AD, Moskowitz GB (2000). Perspective-taking: decreasing stereotype expression, stereotype accessibility, and in-group favoritism. J Pers Soc Psychol.

[30] Locke EA, Latham GP (2002). Building a practically useful theory of goal setting and task motivation. Am Psychol.

[31] Pierce D, Gunn J (2007). Using problem solving therapy in general practice. Aust Fam Physician.

[32] Asimakopoulou K, Newton P, Sinclair AJ, Scambler S (2012). Health care professionals’ understanding and day-to-day practice of patient empowerment in diabetes; time to pause for thought?. Diabetes Res Clin Pract.

